# Advances and prospects in microbial production of biotin

**DOI:** 10.1186/s12934-024-02413-1

**Published:** 2024-05-12

**Authors:** Donghan Ma, Guangqing Du, Huan Fang, Rong Li, Dawei Zhang

**Affiliations:** 1 School of Biological Engineering, Dalian Polytechnic University, Dalian, 116034 China; 2 Tianjin Institute of Industrial Biotechnology, Chinese Academy of Sciences, Tianjin, 300308 China; 3 National Center of Technology Innovation for Synthetic Biology, Tianjin, 300308 China; 4 Key Laboratory of Engineering Biology for Low-Carbon Manufacturing, Tianjin Institute of Industrial Biotechnology, Chinese Academy of Sciences, Tianjin, 300308 China; 5 University of Chinese Academy of Sciences, Beijing, 100049 China

**Keywords:** Biotin, Biosynthetic pathway, Chemical mutagenesis, Metabolic engineering

## Abstract

Biotin, serving as a coenzyme in carboxylation reactions, is a vital nutrient crucial for the natural growth, development, and overall well-being of both humans and animals. Consequently, biotin is widely utilized in various industries, including feed, food, and pharmaceuticals. Despite its potential advantages, the chemical synthesis of biotin for commercial production encounters environmental and safety challenges. The burgeoning field of synthetic biology now allows for the creation of microbial cell factories producing bio-based products, offering a cost-effective alternative to chemical synthesis for biotin production. This review outlines the pathway and regulatory mechanism involved in biotin biosynthesis. Then, the strategies to enhance biotin production through both traditional chemical mutagenesis and advanced metabolic engineering are discussed. Finally, the article explores the limitations and future prospects of microbial biotin production. This comprehensive review not only discusses strategies for biotin enhancement but also provides in-depth insights into systematic metabolic engineering approaches aimed at boosting biotin production.

## Introduction

**Fig. 1:**
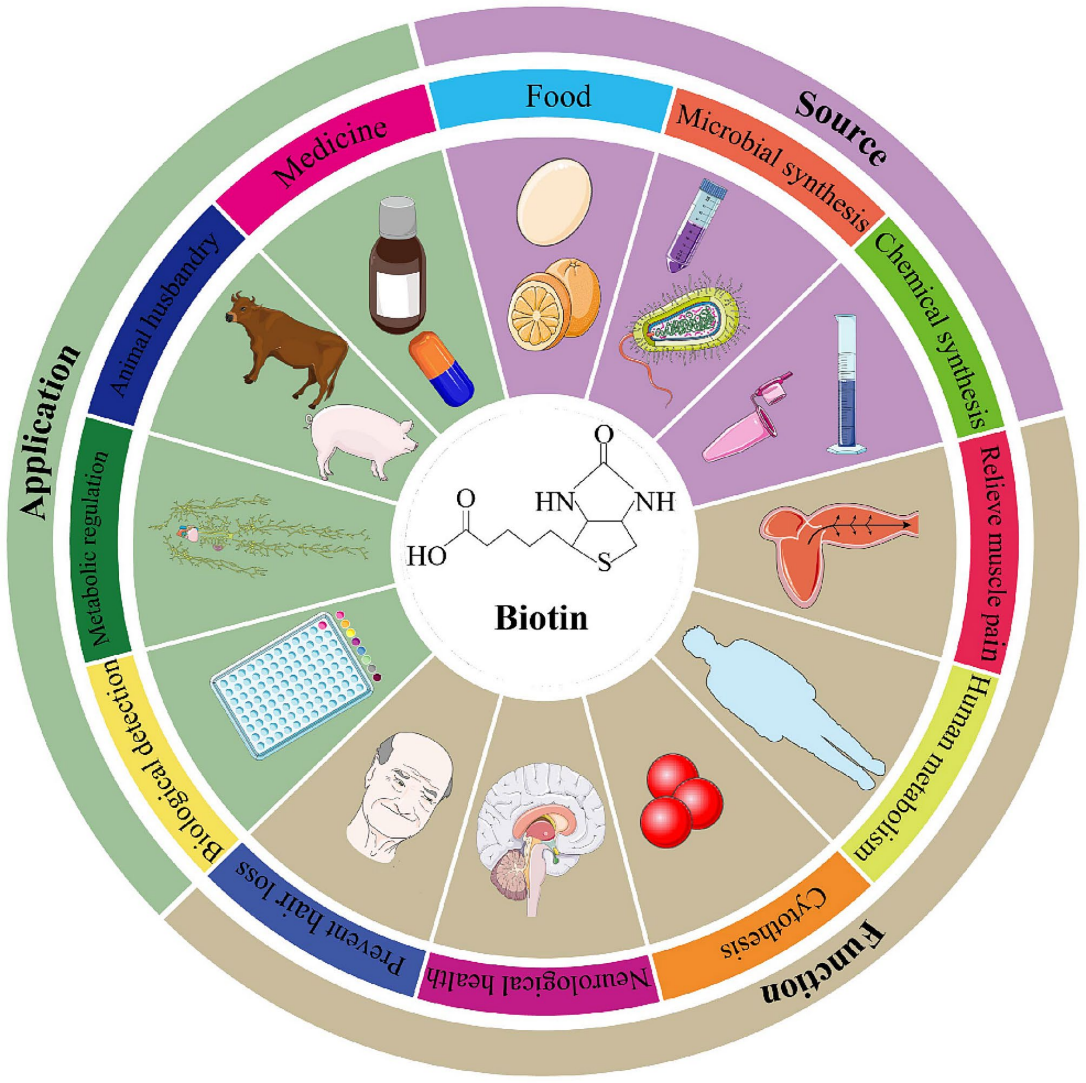
The source, function and industrial application of biotin

Biotin, also known as vitamin B_7_, is essential for maintaining natural human growth, development, and regular physiological functions. Serving as a coenzyme, biotin participates in carboxylation, decarboxylation, and transcarboxylation reactions in biological metabolism [1–3]. Essential carboxylation enzymes, including acetyl-CoA carboxylase (ACC), pyruvate carboxylase (PC), propionyl-CoA carboxylase (PCC), 3-methylcrotonyl-CoA carboxylase (MCC) and urea carboxylase (UC) [4–7]. ACC, PC, PCC, and MCC are widely distributed among most species, while UC is found in certain bacteria and fungi such as *Saccharomyces cerevisiae*. Biotin-dependent carboxylases consist of three domains: biotin carboxyl carrier protein (BCCP), biotin carboxylase (BC), and carboxyl transferase (CT). The BC and BCCP domains of different biotin-dependent carboxylases share the same function, while their CT domains exhibit specificity for different substrates. Most microorganisms and plants can synthesize biotin, while humans and mammals must obtain it through food, intestinal microbiota and products from chemical synthesis [4]. Biotin plays a crucial role in various physiological functions including maintaining human metabolism, cytothesis and neurological health, as well as relieving muscle pain and preventing hair loss [8]. The function relies on its chemical structure, which consists of a bicyclic ring with a tetrahydroimidizalone ring fused to an organosulfur-containing tetrahydrothiophane ring, along with a valeric acid side chain [9]. Biotin attaches to constituent enzymes through an amide linkage between the carboxyl group of the valeric acid side chain and the ε-amine of a specific lysine in the biotin carrier protein [10]. The crystal structure of biotin reveals three chiral carbon atoms and eight stereoisomeric forms, with only the all-cis configuration exhibiting physiological activity [11]. Given its essential role in the carboxyltransfer reactions, biotin deficiency can elevate the risk of skin rash, hair loss and nervous system damage [12]. Usually, biotin deficiency is rare in healthy individuals with a balanced diet, as daily biotin requirements are relatively low (40 µg/d). Mutations in the genes encoding biotinidase (BTD) or holocarboxylase (HLCS) synthetase can lead to Multiple Carboxylase Deficiency (MCD) [13]. Early-stage MCD can be treated with biotin supplementation, but if left untreated, it may cause metabolic disorders, irreversible damage to the nervous system, and even death [14]. Therefore, biotin is an indispensable nutrient for human health, with wide-ranging applications in the fields of biological detection, metabolic regulation, animal husbandry, medicine as well as cosmetics (Fig. 1). Specifically, the medical applications of biotin primarily stem from its pharmacological effects and influence on the immune system. As a coenzyme for carboxylase enzymes, it is essential for enzymes during gluconeogenesis, fatty acid synthesis, and oxidation. Consequently, biotin can lower blood sugar and regulate lipid levels [15], improving diabetic conditions. Additionally, it promotes immune organ growth and enhances the expression of proliferating cell nuclear antigen genes, boosting B lymphocyte conversion efficiency in the blood. For cosmetics application, biotin plays a critical role in the normal growth and skin cell repair [16].

The industrial production of biotin relies on chemical synthesis. Sternbach and Goldberg introduced the chemical synthesis pathway for biotin production using fumaric acid as the raw material in 1949, which was named the Sternbach pathway [17]. This pathway has various advantages including strong stereospecificity and high product yield, laying the foundation for industrial biotin production. However, despite these advantages, this route also had some notable drawbacks, such as the use of toxic reagents, complex procedures and substantial equipment investments [18]. In 2001, a new process for the asymmetric synthesis of biotin emerged, reducing the biotin synthesis pathway from conventional 11 steps to 8 steps, which resulted in lower production cost [19]. This innovation addressed dependence of China on imported biotin, leading to a significant drop in biotin price. Now, China stands as the major producer of biotin, with the global biotin market reaching 1.7 billion RMB in 2020. Given the escalating demand for biotin, the conventional chemical synthesis method is unsustainable due to high production costs and the environmental concerns.

The construction of microbial cell factories emerges as a promising approach for large-scale and economically feasible production of biotin. Many microorganisms, such as *Bacillus subtilis*, *Bacillus sphaericus*, *Escherichia coli*, *Serratia marcescens*, *Kurthia* sp., and *Pseudomonas mutabilis*, can synthesize biotin [20]. The genetics of biotin biosynthesis in these microbes have been elucidated through gene mutation or complementation studies, revealing that the biotin synthesis genes are organized into one or two operons. In *E. coli*, the *bio* operon consists of a rightward transcription unit encompassing genes *bioB*, *bioF*, *bioC* and *bioD*, and a leftward transcription unit including gene *bioA* with an additional open reading frame *orfX* [21, 22]. The sixth separate gene *bioH* is located in a distinct position in the genome [23]. In *B. sphaericus*, the *bio* genes are organized into two distinct operons within the chromosome, whereas *B. subtilis* consolidates all *bio* genes into a single transcriptional unit [24, 25]. Additionally, there are some biotin auxotrophic strains with incomplete biotin synthesis genes. For example, *bioA*, *bioD* and *bioB* exist in the genome of *Corynebacterium glutamicum* and *Sinorhizobium meliloti*, while *bioF*, *bioW* or *bioCH* are absent [26, 27]. Generally, the biotin biosynthesis genes are tightly regulated as biotin is an expensive molecule and 20 ATP equivalents were required to synthesize one biotin [28]. As a result, biotin production is typically restricted in natural wild-type strains.

The advanced progresses in genome editing technology and synthetic biology have made it viable to construct microbial cell factories for efficient production of biotin. Previous reviews predominantly focused on biotin synthesis pathways, regulation, and physiological functions. This review offers a comprehensive summary of recent research progress in developing strains for hyper-biotin production, utilizing chemical mutagenesis and metabolic engineering methods, as well as the innovative strategies for further enhancing biotin production.

### Biotin biosynthesis pathway

**Fig. 2:**
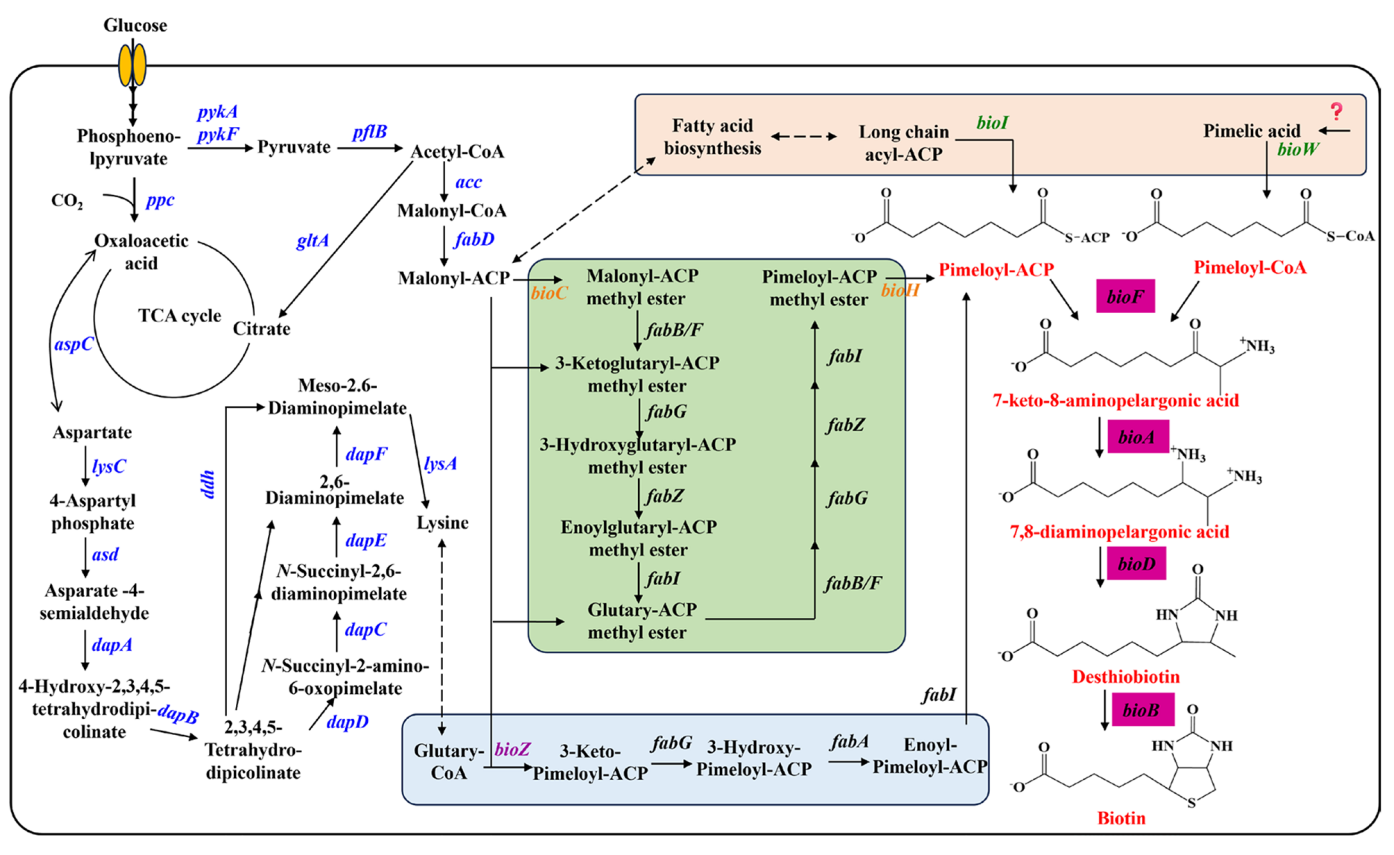
Metabolic pathway of biotin using different precursors. The precursors synthesized by the BioC/BioH, BioW/BioI and BioZ pathways are indicated with light green, pinkish, and light blue backgrounds. *pykA*: pyruvate kinase; *pykF*: pyruvate kinase; *pflB*: formate C-acetyltransferase; *ppc*: phosphoenolpyruvate carboxylase; *acc*: acetyl-CoA carboxylase; *gltA*: citrate synthase; *aspC*: aspartate aminotransferase; *lysC*: aspartate kinase; *asD*: aspartate-semialdehyde dehydrogenase; *dapA*: 4-hydroxy-tetrahydrodipicolinate synthase; *dapB*: 4-hydroxy-tetrahydrodipicolinate reductase; *dapC*: *N*-succinyldiaminopimelate aminotransferase; *dapD*: 2,3,4,5-tetrahydropyridine-2,6-dicarboxylate *N*-succinyltransferase; *dapE*: succinyl-diaminopimelate desuccinylase; *dapF*: diaminopimelate epimerase; *ddh*: meso-diaminopimelate dehydrogenase; *lysA*: diaminopimelate decarboxylase; *fabB*: 3-oxoacyl-[acyl-carrier-protein] synthase I; *fabF*: 3-oxoacyl-[acyl-carrier-protein] synthase II; *fabD*: [acyl-carrier-protein] S-malonyltransferase; *fabG*: 3-oxoacyl-[acyl-carrier protein] reductase; *fabZ*: 3-hydroxyacyl-[acyl-carrier-protein] dehydratase; *fabI*: enoyl-[acyl-carrier protein] reductase I; *bioC*: malonyl-CoA O-methyltransferase; *bioH*: pimeloyl-[acyl-carrier protein] methyl ester esterase; *bioZ*: 3-oxoacyl-[acyl-carrier-protein] synthase III; *bioI*: pimeloyl-[acyl-carrier protein] synthase; *bioW*: 6-carboxyhexanoate–CoA ligase; *bioF*: 8-amino-7-oxononanoate synthase; *bioA*: 7,8-diaminopelargonic acid synthase; *bioD*: dethiobiotin synthase; *bioB*: biotin synthase

The biotin synthesis pathway is highly conserved, with microorganisms and plants sharing similar routes for biotin synthesis. Currently, research on the biotin synthesis pathway in microorganisms is more extensive, while the synthesis pathway in plants is still in its early stages. Most microorganisms possess the ability to carry out de novo synthesis of biotin through a two-stage biosynthetic pathway (Fig. 2). The initial stage involves the synthesis of a pimelate moiety, which contributes the majority of carbon atoms to the biotin backbone. The second stage involves the assembly of a ureido ring fused to a tetrathiophene ring. Three pathways for the synthesis of pimelate moiety have been identified, including the pathways BioC/BioH, BioI/BioW, and BioZ/CaiB. In the following section, we will provide an overview of the two-stage biosynthetic pathway of biotin.

### Biosynthesis of pimelate moiety

BioC, a *S*-adenosyl-L-methionine (SAM)-dependent methyltransferase, is highly conserved in the Proteobacteria phylum [29]. The strain with BioC deletion can only grow in medium with biotin supplemented, affirming the indispensability of this enzyme for biotin synthesis [30]. *E. coli* utilizes the BioC enzyme to methylate the ω-carboxyl group of malonyl-CoA for synthesis of malonyl-CoA methyl ester, which condenses with malonyl-ACP to form stable intermediate 3-oxoglutaryl-ACP methyl ester [31]. This intermediate undergoes standard fatty acid reductase-dehydratase-reductase reactions to convert the 3-oxo group into a methylene group. The C5 product, glutaryl-ACP methyl ester, is elongated through another round of reductase-dehydratase-reductase reactions, yielding the C7 product pimeloyl-ACP methyl ester. The methyl ester is hydrolyzed to expose the ω-carboxyl group and yield the product pimeloyl-ACP. The crystal structure and biochemical characteristic of BioC have not been reported because it is highly prone to form inclusion bodies under overexpression conditions in *E. coli* [32]. The BioC of *B. cereus* is the only one reported to achieve soluble expression in *E. coli*, exhibiting high activity in converting malonyl-ACP to its methylated form and blocking fatty acid synthesis [29]. In contrast, the crystal structure of BioH has been elucidated, revealing it as a monomeric protein with a molecular weight of 28.5 kDa. BioH belongs to the α/β hydrolase enzyme family and has a classical catalytic center composed of Ser-His-Asp [33, 34]. During evolution, seven distinct pimeloyl-ACP methyl ester esterases have been identified, including BioH, BioG, BioJ, BioK, BioV, BtsA and BioU [35]. A hydrolase discovered in archaea also possesses a Ser-His-Asp triad catalytic center and is capable of performing the catalytic function of BioH [36]. The BioCH pathway is a crucial aspect of biotin biosynthesis, playing a key role in the formation of the biotin precursor pimeloyl-ACP. Understanding the BioCH pathway is essential for metabolic engineering and synthetic biology efforts aimed at enhancing biotin production in various organisms.

In *B. subtilis*, the synthesis of the pimelate moiety involves two pathways: the BioW pathway for pimeloyl-CoA synthesis and the BioI pathway for pimeloyl-ACP synthesis [37]. BioI, identified as a cytochrome P450 enzyme, exhibits the ability to cleave long-chain fatty acids and fatty acyl-ACPs to form pimeloyl-ACP [38]. The cleavage of acyl chain C-C bond by BioI needs three rounds of oxidation performed by Fe(v)-oxo species [39]. Unexpectedly, *B. subtilis* is unable to utilize pimeloyl-ACP for biotin synthesis due to the lack of activity of its BioF enzyme toward this precursor [40]. Therefore, the absence of BioI does not significantly impact biotin synthesis or microbial growth in *B. subtilis* [41]. Conversely, deletion of BioW leads to the biotin deficiency phenotype, indicating that BioW is essential for biotin synthesis [42]. BioW is a kind of acyl-CoA synthetase without the typical sequence motifs found in this well-studied enzyme family [43, 44]. It catalyzes condensation of pimelic acid with ATP to form the intermediate pimeloyl-AMP, and followed by thioesterification with CoA to release AMP and form pimeloyl-CoA. The crystal structures of BioW from *B. subtilis* and *A. aeolicus* reveal that they have high consistency in enzyme structures [43, 44]. Considering the predominant role of BioW in biotin synthesis, its catalytic substrate, pimelic acid, is theoretically generated through the metabolic pathways of the wild-type strain. However, this pathway remains unknown in *B. subtilis* or any other strains. Investigating the synthesis pathway of pimelic acid is also an important strategy for improving the supply of pimelate moiety for improved biotin production.

The BioZ synthetic pathway for the precursor pimeloyl-ACP has been identified in *Agrobacterium tumefaciens*, *Brucella melitensis*, and *Sinorhizobium fredii* [45, 46]. Their genomes lack the *bioC*/*bioH* or *bioW* genes, while contain a *bioZ* gene with higher homology to FabH, which is involved in fatty acid synthesis [47]. Deletion or mutation of BioZ resulted in biotin auxotrophy, while expression of BioZ in BioC or BioCH deleted strain enables normal growth in media without biotin supplementation, demonstrating that BioZ functions in the synthesis of biotin precursor [45, 47]. BioZ catalyzes a Claisen condensation reaction between glutaryl-CoA and malonyl-ACP to form 3-ketopimeloyl-ACP. This intermediate enters the fatty acid synthesis pathway, undergoing reduction, dehydration, and reduction reactions to produce pimeloyl-ACP [48]. The substrate for the synthesis of glutaryl-CoA, likely derived from glutamate conversion, is an essential step in this pathway. In *A. tumefaciens*, the type III acyl-CoA transferase CaiB can transfer the CoA moiety from succinyl-CoA to glutamate to form glutaryl-CoA [49].

### Biotin biosynthesis from pimelate moiety

The second stage of biotin synthesis is a highly conserved process starting from the precursor pimelate moiety, sequentially catalyzed by the enzymes BioF/BioA/BioD/BioB. The pimelate moiety contributes two carbon atoms to form its rings and five carbon atoms to from the valeric acid side chain. The remaining three carbon atoms are provided by L-alanine and CO_2_. The sulfur atom is provided by the [Fe-S] cluster, while the two nitrogen atoms come from L-alanine and SAM or lysine.

The initial step in assembling the double ring structure of biotin involves the conversion of pimeloyl-ACP or pimeloyl-CoA to 7-keto-8-aminopelargonic acid (KAPA), which catalyzed by 8-amino-7-oxononanoate synthase BioF in both microorganisms and plants [30, 50]. BioF is a pyridoxal phosphate (PLP)-dependent enzyme belonging to the aminotransferase family [51]. Crystal structures of BioF in *E. coli* and *M. smegmatis* reveal that BioF functions as a dimer, with each monomer comprising three domains: a N-terminal domain, a central core domain and a C-terminal domain [49, 51–53]. This reaction introduces two carbon atoms and one nitrogen atom from alanine, converting the 7 C precursor into a 9 C compound. The substrate specificities of BioF are different between *E. coli* and *B. subtilis*. *E. coli* can utilize both pimeloyl-ACP and pimeloyl-CoA as substrates, while *B. subtilis* can only use pimeloyl-CoA for KAPA synthesis [40]. In other words, *B. subtilis* cannot employ the product synthesized by BioI to produce biotin. A plausible explanation is that BioI is only present in a limited number of microorganisms, and its pathway may have been obstructed during long-term evolution. The BioF protein is also identified in various thermophiles with higher thermostability, facilitating its application for microbial biotin production at elevated temperatures [54].

The second step in biotin biosynthesis involves the transamination of KAPA to 7,8-diaminopelargonic acid (DAPA) [55–57]. This reaction is catalyzed by DAPA synthase BioA, an aminotransferase using PLP as a cofactor [58, 59]. BioA functions as a homodimer and catalyzes the transfer of the amino group of SAM to introduce the second nitrogen atom into the KAPA [56]. It is known to be the only aminotransferase utilizing SAM as the amino donor, which is typically used as methyl donor in product biosynthesis [55, 60]. Kinetics studies have demonstrated that the reaction catalyzed by the BioA enzyme follows a ping-pong mechanism, and its activity is significantly inhibited by the substrate KAPA [61, 62]. Unlike other bacterial strains, *B. subtilis* utilizes L-lysine as the amino donor in the reaction catalyzed by BioA, possibly because its intracellular synthesis is stronger than that of SAM [63]. However, in *B. subtili*s, the *K*_m_ value of BioA for the amino donor L-lysine is much higher than that of BioA in *E. coli* for SAM [60, 62]. Consequently, the KAPA to DAPA conversion catalyzed by BioA is the rate-limiting step in biotin biosynthesis in *B. subtilis*. The addition of exogenous L-lysine can significantly enhance the rate of DAPA production [63]. Biotin biosynthesis is essential for survival of pathogenic bacteria such as *M. tuberculosis*, making it possible to defeat chronic infections by disrupting the biotin synthesis pathway [64]. BioA emerges as a highly promising target, and several antitubercular agents functioning as BioA inhibitors have been developed [65–67]. This suggests that the inhibition of BioA activity may contribute to the low biotin production in various bacterial strains.

The penultimate step in biotin biosynthesis is catalyzed by dethiobiotin (DTB) synthase BioD, which converts DAPA into DTB [68]. This reaction involves the introduction of a carbon atom between the N7 and N8 nitrogen atoms of DAPA to form a ureido ring [68]. The catalytic process involves three steps, including the formation of N7-carbamate, carbamic-phosphoric acid anhydride and finally the closure of the ureido ring. The crystal structures of BioD from *E. coli* and *M. tuberculosis* have been determined [69–71]. The BioD homodimer consists of seven β-fold structures, each connected by α-helices, and the active site with DAPA and ATP binding has been identified [70]. Similar to BioA, BioD has been used as a promising target for the development of antibacterial agents [72, 73].

The final step in biotin synthesis involves the insertion of a sulfur atom between C6 and C9 of DTB via a radical-based mechanism to form the thiazole ring [74]. This reaction is catalyzed by biotin synthase BioB, a member of the SAM radical enzyme family with a conserved iron-sulfur cluster binding sequence [74–76]. The crystal structure of BioB demonstrates it functions as a homodimer, relying on two iron-sulfur clusters: an unstable [4Fe-4S]^2+^ cluster and a stable [2Fe-2S]^2+^ cluster [76]. The BioB catalytic reaction begins with an electron transfer from reduced flavodoxin to SAM, leading to the synthesis of methionine and 5’-deoxyadenosine radical. The 5’-deoxyadenosyl radical cleaves a C-H bond and abstracts a hydrogen atom at the C9 of DTB. This step generates a carbon radical at C9 of DTB, which attacks the bridging sulfur of the [2Fe-2S]^2+^ cluster, forming an intermediate with DTB linked to [2Fe-2S]^2+^. Subsequently, the hydrogen atom at C6 of DTB is abstracted by another 5’-deoxyadenosyl radical, forming a carbon radical that attacks the same sulfur atom of the [2Fe-2S]^2+^ cluster. Finally, the sulfur atom from [2Fe-2S]^2+^ cluster is inserted between C6 and C9 atoms, forming the tetrathiophene ring [77, 78]. This reaction consumes two molecules of SAM to yield two molecules of methionine and 5’-deoxyadenosine. Methionine can be recycled in the reaction catalyzed by the SAM synthase, while 5’-deoxyadenosine has been demonstrated to be a toxic byproduct for bacterial cells [75, 79–81]. To alleviate byproduct inhibition, a 5’-methylthioadenosine/*S*-adenosyl-homocysteine nucleosidase is required for 5’-deoxyadenosine degradation [82]. Additionally, the regeneration of the [2Fe-2S]^2+^ cluster by the Isc or Suf iron-sulfur cluster assembly system is another bottleneck issue in biotin synthesis [83, 84]. Despite extensive research on the structure and catalytic mechanism of BioB in the four conserved steps of biotin synthesis, there is currently limited exploration aimed at improving its catalytic activity. Consequently, the conversion of DTB to biotin catalyzed by BioB remains the most significant bottleneck in constructing efficient biotin cell factories.

### Regulation of biotin biosynthesis

**Fig. 3:**
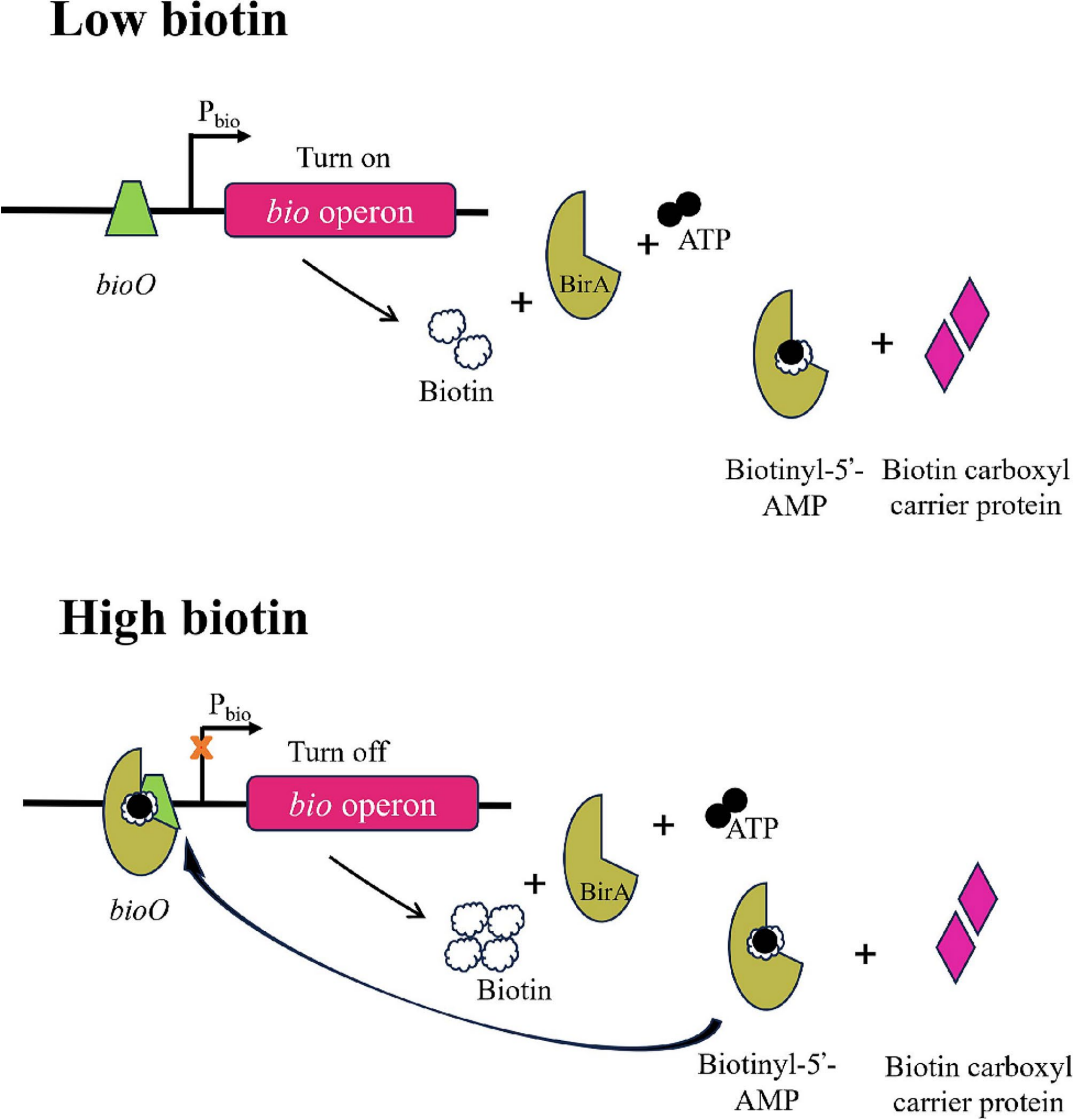
The regulation of biotin synthesis by BirA. When the biotin titer is low, the *bio* operon keeps at transcriptionally active state, and when the biotin is accumulated, derepression of *bio* operon transcription is observed

During microbial growth, cells can obtain biotin through two mechanisms: uptake from the environment or de novo synthesis [85]. Typically, the genes involved in biotin synthesis are organized in an operon, ensuring simultaneous expression for biotin production [86]. In the presence of environmental biotin, microbial cells tend to directly uptake exogenous biotin [87]. The synthesis of biotin is a highly energy-consuming process, requiring 20 mol equivalents of ATP for 1 mol of biotin [28]. Additionally, the lack of cofactor SAM and the relatively low catalytic activity of BioB contribute to the reluctance of bacterial strains to synthesize biotin [88]. Therefore, biotin synthesis is tightly regulated in microbial cells. Currently, regulatory proteins such as BirA, BioR and BioQ are known to control biotin synthesis in various bacterial strains [89–91]. There are three types of BirA proteins depending on their different domain architectures. The type I BirA only contains catalytic domain that is essential for protein biotinoylation. In typeII BirA, a DNA binding domain in the N-terminus plays a role in inhibition of gene expression. These two types of regulators are widely present in microorganisms. The type III BirA presented in eukaryotes and mammals contains a long N-terminal extension, which can increase the specificity for the biotinoylated target. The type II BirA is the first regulatory factor discovered in *E. coli* as a bifunctional protein. In the presence of ATP, BirA binds biotin and ATP to form a BirA-biotinyl-5’-adenylate intermediate. When cellular biotin levels are low, the intermediate transfers the biotinyl moiety to the biotin carboxyl carrier protein (BCCP) for protein biotinylation. On the other hand, it binds to the *bioO* sequence of biotin operon to inhibit the expression of biotin synthesis genes (Fig. 3) [92]. In this way, it ensures that when the biotin is in excess for normal cell growth, cells halt biotin synthesis to alleviate cellular burden. This type is also found in bacteria such as *B. subtilis* and *Staphylococcus aureus* [93]. Differently, the BirAs in these two strains are also involved in regulating biotin uptake, indicating that they functions are somewhat different among various bacteria [94]. In *B. subtilis* and *S. aureus*, BirA represses the expression of biotin transporter-encoding gene *bioY* and its downstream genes *yhfS* and *yhfT* by binding to their *bioO* sequence [95]. Additionally, some strains contain two BirA proteins. For example, in *F. novicida*, there is a BplA protein lacking DNA binding functionality and essential for cell growth besides Type II BirA [96].

Some bacterial strains lack any type of BirA for protein biotinoylation. Instead, they contain BioR and BioQ, which perform the same function as BirA [97, 98]. In some bacteria like α-proteobacteria, the GntR family transcriptional regulator BioR can perform the transcriptional regulatory role of BirA [90]. Biotin metabolism mediated by BioR exhibits complex regulation. For instance, in *Brucella melitensis*, BioR recognizes sequences upstream of genes encoding *bioR*, *bioY* and *bioB*. Experimental evidence suggests that BioR not only suppresses the transcription of biotin synthesis genes but also possesses self-regulation and mediates the expression of biotin transport proteins [97]. In actinomycetes, the TetR family transcriptional regulator BioQ can regulate biotin synthesis in a similar manner with BioR [91, 99]. To enhance biotin production, the primary strategy is to remove the DNA binding functionality while retaining their catalytic function of these regulatory proteins.

### Strategy for improved biotin production

Currently, the wild-type strains reported in the literature exhibit very low initial biotin yields, typically below 10 µg/L. Strain modification is necessary to increase biotin production and various strategies have been explored. The main approach involves chemical mutagenesis and metabolic engineering of microorganisms to optimize the biosynthetic pathway for increased biotin production. The following section focuses on introducing the specific experimental designs and the impacts on biotin synthesis of the two strategies.

### Chemical mutagenesis

Before the widespread application of efficient gene editing technologies, mutagenesis methods are mainly used to enhance biotin production. Analogs of biotin are tested for rapid screening of strains with higher biotin production. Two biotin analogs actithiazic acid (ACM) and 5-(2-thienyl) valeric acid (TVA), have been demonstrated to be the most effective [100–102]. The summary of improved biotin production by chemical mutagenesis is shown in Table 1. By treating *B. sphaericus* with chemical mutagens *N*-methyl-*N*’-nitro-*N*-nitrosoguanideine (NTG), ACM and TVA resistant mutants were selected and evaluated for biotin production. A mutant strain showed a 11.9-fold improvement in biotin titer compared to the wild-type strain and accumulated 9.5 mg/L of biotin [103]. Sakurai et al. [104] employed the same biotin analogs and mutagens, and screened a mutant strain *Serratia marcescens* SB412, which produced biotin at a titer of 20 mg/L. Further expressing the biotin synthesis operon *bioABFCD* in the SB412 strain significantly increased the biotin titer to 200 mg/L [105]. To reinforce the supply of cofactor SAM, SAM structural analogs ethionine and *S*-2-aminoethylcysteine were used for a second round of mutagenesis on strain SB412. A mutant strain ETA23 was obtained with a biotin titer of 33 mg/L [106]. By transforming the pLGM304P plasmid carrying the *bioABFCD* operon into ETA23, biotin production reached up to 250 mg/L in shake flask fermentation and 500 mg/L in fed-batch fermentation [106]. By optimizing the fermentation conditions, the maximum biotin production of 600 mg/L can be achieved [111]. To maintain strain stability during fermentation, the biotin biosynthetic genes were integrated into the genome of strain ETA23. The resulting strain produced biotin at 120 mg/L in the medium containing sucrose and urea [107]. The construction of these high-yield biotin-producing bacterial strains demonstrates that *S. marcescens* is a potential chassis strain for biotin production. The genus *Kurthia* is another type of microbial strain that can be used for constructing biotin cell factories. A mutant strain named *Kurthia* sp. 538-51F9 with resistance to α-methyl dethiobiotin (MeDTB) was obtained with biotin titer of 126 mg/L in flask fermentation [108]. *E. coli* was also employed as the host strain for chemical mutagenesis. After two rounds of TVA- and ACM-resistant screening, a resulting mutant strain exhibited a biotin titer of 4.5 mg/L [109]. Enhancing the cellular level of SAM is advantageous for accelerating biotin biosynthesis. The mutants resistant to threonine analogue theoretically have a higher SAM metabolic flux. By selecting threonine analogue-resistant mutants, a high-yield biotin-producing strain with biotin titer of 970 mg/L was obtained, representing the highest level reported in *E. coli* [110, 111]. *Pseudomonas mutagenesis* can supply a high level of intracellular NADPH and exhibit a prominent advantage in synthesizing NADPH-consumed products [112]. Meanwhile, it can tolerate high concentrations of biotin and lack a degradation pathway, making it a promising strain for biotin production [113]. Four biotin analogs, including biotin methyl ester, 4-aminobenzoic acid, biotin p-nitrophenyl and diaminobiotin, were used for screening biotin analog-resistant mutants. In combination with the overexpression of the biotin biosynthetic genes *bioABFHCD* from *Pseudomonas aeruginosa*, efficient biotin production was achieved with its titer increased to 15 g/L [114]. Such high biotin production has presented the potential for industrial-scale production; however, there have been no subsequent reports on this high-yield strain. In summary, employing chemical mutagenesis for initial screening and subsequently reinforcing the biotin biosynthetic pathway represent a viable approach for enhancing biotin production.

**Table 1:** The summary of improved biotin production by chemical mutagenesis

Host	Biotin analogs	Mutagenic agent	Genotype	Titer (mg/L)	Reference
*B. sphaericus*	ACM, TVA	NTG	-	9.5^S^	[103]
*S. marcescens*	ACM, TVA	NTG	-	20^S^	[104]
*S. marcescens*	-	-	*bioABFCD*	200^S^	[105]
*S. marcescens*	Ethionine; S-2-aminoethy-lcysteine	NTG	*-*	33^S^	[106]
			*bioABFCD*	250^S^	
				500^FB^	
*S. marcescens*	-	-	*bioABFCD*	600^FB^	[111]
*S. marcescens*	Ethionine; S-2-aminoethy-lcysteine	NTG	*bioBFCD* (Chromosome)	120^S^	[107]
*Kurthia* sp.	Acidomycin; TVA; α-methyl dethiobiotin; 2-methyl acidomycin; amiclenomycin; bisnorbiotinol	NTG	*-*	126^S^	[108]
*E. coli*	ACM, TVA	NTG	*-*	4.5^S^	[109]
*E. coli*	Threonine analogue	NTG	*bioABFCD*	970^FB^	[110, 111]
*P. mutagenesis*	Biotin methyl ester; biotin pnitrophenyl; 4-amido-benzo-ic acid; diamino biotin	NTG	*bioABFHCD*	15 000^B^	[114]

^#^ S: shake flask; B: batch; FB: fed-batch; MP: medium optimization

### Metabolic engineering

The metabolic engineering strategies used to enhance product production mainly include relieving feedback repression, strengthening metabolic pathways, increasing precursor and cofactor supply. For example, in the production of L-valine, a > 14-fold increase in the L-valine titer can be obtained by relieving transcriptional repression and strengthening metabolic pathways [115]. Li et al. used multiple metabolic engineering strategies including relieving feedback inhibition, increasing precursor, and enriching the cofactor NADPH pool to improve methionine production. The resulted strain can produce 6.85 g/L of methionine with an increase of more than 30-fold [116]. Liu et al. conducted rational design of the rate-limiting enzymes PdxA and PdxJ in the vitamin B6 synthesis pathway to reinforce the metabolic pathway of the product. As a result, the vitamin B6 titer increased from 0.05 mg/L to 1.4 g/L [117]. Kim et al. employed a quorum sensing (QS)-dependent manner to enhance the pool of CoA-derived precursors for neoaureothin production [118]. The productivity of engineered strain increased by up to 4-fold. These examples suggest that these metabolic engineering approaches can significantly enhance production of various products.

For microbial production of biotin, the used metabolic engineering approaches and their effects on biotin improvement were show in Table 2. *E. coli* is the most extensively studied host strain for improving biotin production. Plasmids carrying the *bioB* gene from *B. sphaericus* were transformed into *E. coli* to evaluate their impact on biotin production. The engineered strain exhibited a remarkable increase in biotin titer to 16 mg/L, underscoring the beneficial effect of *bioB* gene overexpression on biotin synthesis [119]. In comparison to expressing the solitary *bioB* gene, introducing the entire biotin synthesis operon *bioXWF* and *bioDAYB* from *B. sphaericus* IFO3525 resulted in more biotin production of 27.0 mg/L and 45.0 mg/L in batch and fed-batch fermentations, respectively [120]. Modifying the biotin biosynthesis operon, rather than directly expressing heterologous *bio*-operons, can also effectively enhance biotin biosynthesis. The reorganization and expression of the *E. coli* biotin operon with a robust *tac* promoter resulted in an increased biotin titer of 35 mg/L [121]. When expressing this modified operon in *Grobacterium/Rhizobium*, the biotin titer increased to 110 mg/L in fed-batch fermentation [121]. IscR is a global transcriptional regulator responsible for the regulation of iron-sulfur cluster assembly. A point mutation of IscR alleviated the toxic effect of BioB overexpression and increased the conversion efficiency of DTB to biotin by 2.2-fold, demonstrating the assemble of iron-sulfur cluster is a potential bottleneck in biotin synthesis [122]. Wei et al. [123] expressed the biotin synthesis operon *bioBFHCDA* and introduced the genes *bioW* from *B. subtilis* and *sam2* from *S. cerevisiae*, the recombinant strain PM02 achieved an increased biotin titer of 12.8 mg/L. After medium optimization, the biotin production further increased to 208.7 mg/L in fed-batch fermentation, which is the highest level ever reported in *E. coli* strains. [123]. In comparison to *E. coli, B. subtilis* emerges as a more suitable chassis cell for biotin synthesis, as it is internationally recognized as a safe strain [124]. Ohsawa et al. [119] expressed the *bioB* gene of *B. sphaericus* in *B. subtilis*, resulting in an increase in the biotin production from 2 mg/L to 15 mg/L. Overexpression of the biotin operon rather than single *bioB* gene did not lead to a higher biotin production, confirming the vital role of BioB in biotin synthesis [125]. Wu et al. [126] replaced the promoter of the biotin biosynthetic operon with the *groE* promoter. A biotin titer of 5 mg/L was achieved by inducing expression with gluconate. Van Arsdell et al. [63] used the constitutive promoter SP01-15 to express its biotin synthesis operon. The recombinant strain could produce a large amount of KAPA with the addition of pimelic acid in medium. However, only trace amounts of DTB and biotin were synthesized, demonstrating that BioA, which catalyzes the synthesis of DAPA from KAPA, may be the rate-limiting enzyme. Further overexpression of BioA with a strong promoter did not significantly change the amounts of DAPA and DTB. Finally, DTB production significantly increased to 750 mg/L by externally adding lysine, which is the amino donor of the reaction catalyzed by BioA. This study provides an effective method for significantly increasing DTB production, but the bottleneck problem of the low conversion rate from DTB to biotin has not been resolved. As mentioned above, *P. mutabilis* is reported as the most suitable strain for biotin production. Zhao et al. [127] developed a CRISPR-Cas12a system for *yigM* deletion, *birA* repression and *bioWIAFD* integration in *P. mutabilis.* The constructed strain produced 4.7-fold more biotin compared with the wild type strain with a titer of 177.4 µg/L. Via multiple metabolic engineering strategies and process engineering, the constructed *P. mutabilis* strain can produce biotin at a titer of 271.9 mg/L [128]. Some other strains, such as *Agrobacterium/Rhizobium*, *Sphingomonas* sp. and *C. utilis*, were also used as parental strains for improved biotin production [121, 129, 130]. It can be seen that the main employed strategy to enhance biotin production through metabolic engineering involves relieving the negative regulation of biotin synthesis and overexpressing exogenous biotin synthesis operon or BioB. Biotin production in shake flask fermentation remains at a relatively low level, and the increase in production is mainly achieved through medium and fermentation optimizations. The enzymes catalyzing the five-step reactions from pimelic acid to biotin exhibit very low catalytic activity, which is the main bottleneck restricting biotin production enhancement. In the future work, the advanced AI technology can be used to predict and screen biotin synthesis enzymes with high catalytic efficiency. Then, the selected enzymes with different expression levels can be assembled with MetClo technique for coordinated gene expression to boost overall metabolic flux. Through these methods, it is hopeful that biotin production can be significantly increased.

**Table 2:** The summary of improved biotin production by metabolic engineering

Host	Characteristics*	Titer (mg/L) ^#^	Reference
*E. coli*	Overexpression of *bioB-Bs*	16^S^	[119]
*E. coli*	Overexpression of *bioXWF-Bs* and *bioDAYB-Bs*	27.0^B^; 45^FB^	[120]
*E. coli*	Overexpression of *bioBFCDA*	1.4^S^; 35^FB^	[121]
*E. coli*	Site mutation of *iscR*	2.0^S^	[122]
*E. coli*	Overexpression of *bioABFHCD-Pp* and *bioW-sam2*	12.8^S^; 208.7^S^	[123]
*B. subtilis*	Overexpression of *bioB-Bs*	15^S^	[119]
*B. subtilis*	Overexpression of *bioWAFDBI-Bs2*	1.1^S^; 8^B^; 16^MP^	[125]
*B. subtilis*	Using *groE* promotor to replace the origin one	5.0^S^	[126]
*B. subtilis*	Overexpression of *bioWAFDBI-ytbQ-Bs2*	5.0^S^	[63]
*P. mutabilis*	Knockout of *yigM*; repression of *birA*; Overexpression of *bioWIAFD-Bs2*	0.18^S^	[127]
*P. mutabilis*	RBS optilization of *bioA* and *bioBFHCD*; Δ*yigM*; *birA*::*EcbirA*; overexpression of *metK*, *mtn* and *bioWIAFD-Bs2*	4.6^S^;271.9^S^	[128]
*Agrobacterium/Rhizobium*	Overexpression of *bioBFCDA*	110^FB^	[121]
*Sphingomonas* sp.	Overexpression of *bioBFCDA*	66^FB^	[129]
*C. utilis*	Overexpression of *bio2*	1.9^S^	[130]

*Bs: Bacillus sphaericus; Bs2: Bacillus subtilis; Pp: pseudomonas putida

^#^ S: shake flask; B: batch; FB: fed-batch; MP: medium optimization

## Conclusions and future perspectives

Biotin, a vitamin product with significant industrial value, finds wide-ranging applications in animal feed, food additives, cosmetics, and pharmaceuticals. Despite the maturity of chemical synthesis technology, challenges persist regarding chemical pollutant emissions and high raw material costs. Currently, 80% of the biotin market serves to the feed industry, where biotin produced using safe strains can be directly utilized with microbial biomass, leading to significant cost reductions in subsequent separation process. This highlights the ongoing potential for the broad development of microbial biotin production. Traditional mutagenesis and metabolic pathway optimization have made progress in increasing biotin production, a comprehensive understanding of the metabolic network and enhancing key enzymes catalytic activity are imperative for further progress. With rapid advancements of genomics, gene editing technologies, bioinformatics and protein engineering, exploring efficient biotin synthesis enzymes and studying biotin synthesis regulatory mechanisms are now feasible. Rational design or protein-directed evolution techniques can be applied to address the bottleneck issue of low conversion efficiency from DTB to biotin for the rate-limiting enzyme BioB. In addition, the exploration and application of new pathway enzymes, such as BioU [131] and Type II BioB [132, 133], are also important aspects in increasing biotin production. It is anticipated that high-yielding biotin-producing strains can be obtained to replace chemical synthesis methods and facilitate green production of biotin.

## Data Availability

No datasets were generated or analysed during the current study.

## Abbreviations

BTD: Biotinidase
HLCS: Holocarboxylase
MCD: Multiple Carboxylase Deficiency
SAM: *S*-adenosyl-L-methionine
ACC: Acetyl-CoA Carboxylase
PC: Pyruvate Carboxylase
PCC: Propionyl-CoA Carboxylase
MCC: 3-methylcrotonyl-CoA Carboxylase
UC: Urea Carboxylase
BCCP: Biotin Carboxyl Carrier Protein
BC: Biotin Carboxylase
CT: Carboxyl Transferase
KAPA: 7-keto-8-aminopelargonic acid
PLP: Pyridoxal phosphate
DAPA: 7,8-diaminopelargonic acid
DTB: Dethiobiotin
ACM: Actithiazic acid
TVA: 5-(2-thienyl) valeric acid
NTG: N-methyl-N’-nitro-N-nitrosoguanideine
